# Species Tree Inference by Minimizing Deep Coalescences

**DOI:** 10.1371/journal.pcbi.1000501

**Published:** 2009-09-11

**Authors:** Cuong Than, Luay Nakhleh

**Affiliations:** Department of Computer Science, Rice University, Houston, Texas, United States of America; University of Chicago, United States of America

## Abstract

In a 1997 seminal paper, W. Maddison proposed *minimizing deep coalescences*, or MDC, as an optimization criterion for inferring the species tree from a set of incongruent gene trees, assuming the incongruence is exclusively due to lineage sorting. In a subsequent paper, Maddison and Knowles provided and implemented a search heuristic for optimizing the MDC criterion, given a set of gene trees. However, the heuristic is not guaranteed to compute optimal solutions, and its hill-climbing search makes it slow in practice. In this paper, we provide two *exact* solutions to the problem of inferring the species tree from a set of gene trees under the MDC criterion. In other words, our solutions are guaranteed to find the tree that minimizes the total number of deep coalescences from a set of gene trees. One solution is based on a novel integer linear programming (ILP) formulation, and another is based on a simple dynamic programming (DP) approach. Powerful ILP solvers, such as CPLEX, make the first solution appealing, particularly for very large-scale instances of the problem, whereas the DP-based solution eliminates dependence on proprietary tools, and its simplicity makes it easy to integrate with other genomic events that may cause gene tree incongruence. Using the exact solutions, we analyze a data set of 106 loci from eight yeast species, a data set of 268 loci from eight *Apicomplexan* species, and several simulated data sets. We show that the MDC criterion provides very accurate estimates of the species tree topologies, and that our solutions are very fast, thus allowing for the accurate analysis of genome-scale data sets. Further, the efficiency of the solutions allow for quick exploration of sub-optimal solutions, which is important for a parsimony-based criterion such as MDC, as we show. We show that searching for the species tree in the compatibility graph of the clusters induced by the gene trees may be sufficient in practice, a finding that helps ameliorate the computational requirements of optimization solutions. Further, we study the statistical consistency and convergence rate of the MDC criterion, as well as its optimality in inferring the species tree. Finally, we show how our solutions can be used to identify potential horizontal gene transfer events that may have caused some of the incongruence in the data, thus augmenting Maddison's original framework. We have implemented our solutions in the PhyloNet software package, which is freely available at: http://bioinfo.cs.rice.edu/phylonet.

## Author Summary

Inferring the evolutionary history of a set of species, known as the *species tree*, is a task of utmost significance in biology and beyond. The traditional approach to accomplishing this task from molecular sequences entails sequencing a gene in the set of species under consideration, reconstructing the gene's evolutionary history, and declaring it to be the species tree. However, recent analyses of multiple gene data sets, made available thanks to advances in sequencing technologies, have indicated that gene trees in the same group of species may disagree with each other, as well as with the species tree. Therefore, the development of methods for inferring the species tree despite such disagreements is imperative.

In this paper, we propose such a method, which seeks the tree that minimizes the amount of disagreement between the input set of gene trees and the inferred one. We have implemented our method and studied its performance, in terms of accuracy and computational efficiency, on two biological data sets and a large number of simulated data sets. Our analyses, of both the biological and synthetic data sets, indicate high accuracy of the method, as well as computationally efficient solutions in practice. Hence, our method makes a good candidate for inferring accurate species trees, despite gene tree disagreements, at a genomic scale.

## Introduction

Accurate *species trees*, which model the evolutionary histories of sets of species, play a central role in comparative genomics, conservation studies, and analyses of population divergence, among many other applications. Traditionally, a species tree is inferred by sequencing a single locus (gene) in a group of species, its tree, known as the *gene tree*, is reconstructed using a method such as maximum likelihood, and this tree is declared to be the species tree. The underlying assumption is, obviously, that the gene tree and the species tree are identical, and hence reconstructing the former amounts to learning the latter. However, biologists have long recognized that this assumption is not necessarily always valid. Nevertheless, due to limitations of sequencing technologies, this approach remained the standard method until very recently.

With the advent of whole-genome sequencing, complete genomes of various organisms are becoming increasingly available, and particularly important, data from multiple loci in organisms are becoming available. The availability of such data has allowed for analyzing multiple loci in various groups of species. These analyses have in many cases uncovered widespread incongruence among the gene trees of the same set of organisms. Therefore, while reconstructing a gene tree requires considering the process of nucleotide substitution, reconstructing a species tree requires, in addition, considering the process that resulted in the incongruities among the gene trees, so that the species phylogeny is inferred by reconciling these incongruities. In this paper, we address the problem of efficient inference of accurate species trees from multiple loci, when the gene trees are assumed to be correct, and their incongruence is assumed to be exclusively due to (incomplete) *lineage sorting*. We also address the integration of horizontal gene transfer, as a potential cause of gene tree incongruence, into the framework. Let us illustrate the process of lineage sorting and the way it causes gene tree incongruence.

**Figure 1:**
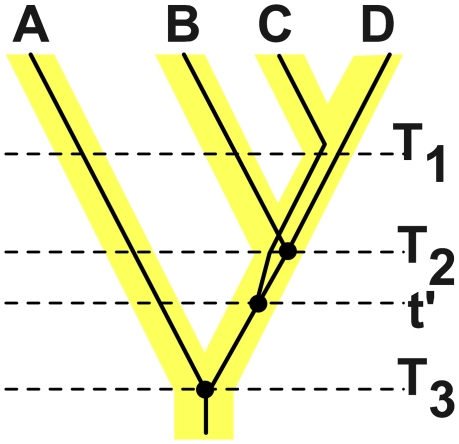
Lineage sorting within the branches of a species tree. Even though *C* and *D* diverged from their most recent common ancestor at time *T*_1_, going back in time one observes that their gene lineage (solid lines) persisted further in the past and *coalesced* at time *t*′, which preceded the speciation time *T*_2_. In this scenario, the gene lineages from *B* and *D* happened to coalesce at time *T*_2_, after *t*′, thus resulting in gene tree (*A*, (*C*, (*B*, *D*))) that disagrees with the species tree (*A*, (*B*, (*C*, *D*))).

From an evolutionary perspective, and barring any recombination, the evolutionary history of a set of genomes would be depicted by a tree that is the same tree that models the evolution of each gene in these genomes. However, events such as recombination break “linkage” among the different parts of the genome, and those unlinked parts may take different paths through the phylogeny, which results in gene trees that differ from the species tree as well as from each other, due to lineage sorting. Widespread gene tree incongruence due to lineage sorting has been shown recently in several groups of closely related organisms, including yeast [1], Drosophila [2], Staphylococcus aureus [3], and *Apicomplexan* [4]. In this case, gene trees need be reconciled *within* the branches of the species tree, as shown in Figure 1.

**Figure 2:**
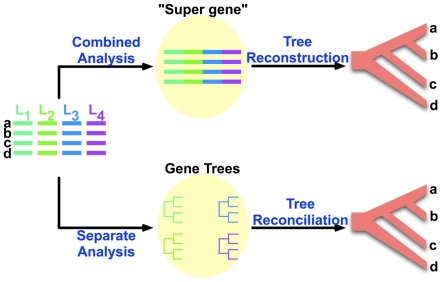
Approaches for inferring species trees. In the combined analysis approach (top), the sequences of the four loci are concatenated, generating one sequence data set, which is then analyzed by any of a host of phylogenetic tree reconstruction methods. In the separate analysis approach (bottom), a gene tree is reconstructed for each locus, and a species tree that reconciles their incongruence is inferred.

A few methods have been introduced recently for analyzing gene trees, reconciling their incongruities, and inferring species trees despite these incongruities. Generally speaking, each of these methods follows one of two approaches: the *combined analysis* approach or the *separate analysis* approach; see Figure 2. In the combined analysis aproach, the sequences from multiple loci are concatenated, and the resulting “supergene” data set is analyzed using traditional phylogenetic methods, such as maximum parsimony or maximum likelihood; e.g., [1]. In the separate analysis approach, the sequence data from each locus is first analyzed individually, and a reconciliation of the gene trees is then sought. One way to reconcile the gene trees is by taking their majority consensus; e.g., [4]. Another is the “democratic vote” method, which entails taking the tree topology occurring with the highest frequency among all gene trees as the species tree. Shortcomings of these methods based on the two approaches have been analyzed by various researchers [5],[6]. Recently, Bayesian methods following the separate analysis approach have been developed [7],[8]. While these methods have a firm statistical basis, they are very time consuming, taking hours and days even on moderate-size data sets, which limits their scalability (for example, the BEST tool of [7] took 800 hours on the yeast data set of [1]).

In [9], Maddison proposed a parsimony-based approach for inferring species trees from gene trees by minimizing the number of extra lineages, or minimizing deep coalesces (MDC). A heuristic for this approach was later described in [10]. In [3], Than *et al.* provided a two-stage heuristic for inferring the species tree under the MDC criterion. However, no exact solutions for computing the MDC criterion exist. In this paper, we provide a formal definition of the notion of extra lineages, first described in [9]. We then present exact solutions—an integer linear programming (ILP) algorithm and a dynamic programming (DP) algorithm—for finding the optimal species tree topology from a set of gene tree topologies, under the MDC criterion (see Methods). Our solutions are based on two central observations: (1) the species tree is a maximal clique in the compatibility graph of the set of species clusters, and (2) quantifying the amount of incongruence between a set of gene trees and a species tree can be obtained by a simple counting of lineages within the branches of the species tree. The accuracy and computational efficiency of these solutions, as we demonstrate, allow for analysis of genome-scale data sets and analysis of large numbers of data sets, such as those involved in simulation studies. Given that MDC is a parsimonious explanation of the incongruence in the data, it is imperative that sub-optimal solutions are considered. The computational efficiency of our solutions allow for a rapid exploration of sub-optimal solutions. Last but not least, these exact solutions allow us to empirically study properties of MDC as an optimality criterion for inferring the species tree. We have implemented both exact solutions in the PhyloNet software package [11].

We reanalyze the *Apicomplexan* data set of [4] (268 loci from eight species), the yeast data set of [1] (106 loci from 8 yeast species), and a large number of synthetic data sets of species/gene trees (up to 2000 loci from 8 species) that we simulated using the Mesquite tool of [12]. For each data set, our method computed the species tree in at most a few seconds (in some cases, it took 0.01 seconds), and produced very accurate species trees, as we show. In the case of the *Apicomplexan* data set, we provide a tree that is slightly different from the one proposed by the authors in [4], and discuss this tree. For the yeast data set, we obtain a tree that is identical to the one proposed by the authors in [1], as well as other studies, such as [7]. In addition to the quality of the species trees and efficiency with which our method inferred them, one advantage of our method is that it can be used in an exploratory fashion, to screen multiple species tree candidates, and study the reconciliation scenarios within the branches of each of them. We illustrate the utility of this capability on the yeast and *Apicomplexan* data sets. Further, for the *Apicomplexan* data set, we illustrate how to screen for possible horizontal gene transfer events using the reconciliation scenarios computed by other methods. Using the synthetic data sets, we study the statistical consistency, as well as convergence rate, of the MDC criterion. We also show that it may be sufficient to consider only the set of clusters induced by the gene trees, which, in practice, may be much smaller than the set of all clusters of species, thus achieving further reduction in computation time. Nonetheless, we present an example to illustrate that, in certain cases, focusing only on the gene tree clusters may result in a sub-optimal species tree under MDC. The computational efficiency of our methods, coupled with the promising properties of the MDC criterion, makes our methods particularly applicable to large, genome-scale data sets.

## Results/Discussion

### Data

**Figure 3:**
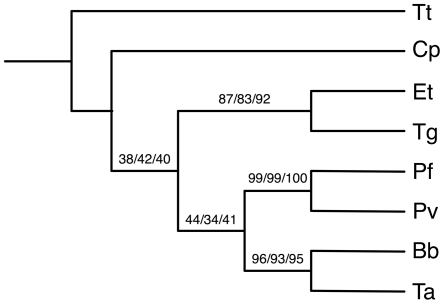
The species tree for the *Apicomplexan* data as inferred using the majority consensus method and reported in [4]. The species *Tt* (*Tetrahymena thermophila*) is the outgroup. The numbers on the tree branches are bootstrap support values based on maximum likelihood, maximum parsimony and neighbor joining methods, respectively.

In this paper, we reanalyze two biological data sets under the MDC criterion: the *Apicomplexan* data set of [4] and the yeast data set of [1]. The *Apicomplexan* data set contains eight species: *Babesia bovis* (Bb), *Cryptospordium pavum* (Cp), *Eimeria tenella* (Et), *Plasmodium falciparum* (Pf), *Plasmodium vivax* (Pv), *Theileria annulata* (Ta), *Toxoplasma gondii* (Tg), and *Tetrahymena thermophila* (Tt). The authors in [4] identified 268 single-copy genes suitable for phylogenetic inference. For each gene, they reconstructed its tree using three methods (maximum parsimony, maximum likelihood, and neighbor joining). Among the 268 gene trees, there were 48 different gene-tree topologies, the most frequent of which appears with about 18% frequency. [4] inferred the species tree using two different methods: the concatenation method and the majority consensus method, both of which produced the same tree, shown in Figure 3, which the author presented as their hypothesis for the species tree of these eight *Apicomplexan* species.

**Figure 4:**
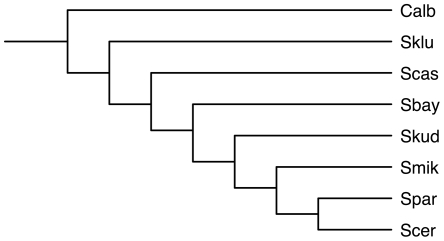
The species tree for the yeast data set as inferred using the concatenation method and reported in [1]. All branches in the tree have 100% bootstrap support values.

The yeast data set contains seven Saccharomyces species *S. cerevisiae* (Scer), *S. paradoxus* (Spar), *S. mikatae* (Smik), *S. kudriavzevii* (Skud), *S. bayanus* (Sbay), *S. castellii* (Scas), *S. kluyveri* (Sklu), and the outgroup fungus *Candida albicans* (Calb). [1] identified 106 genes, which are distributed throughout the *S. cerevisiae* genome on all 16 chromosomes and comprise about 2% of predicted genes. For each gene, they reconstructed its tree using the maximum likelihood and maximum parsimony methods. Among the 106 trees, more than 20 different gene-tree topologies were observed. The authors in [1] inferred the species tree using the concatenation method on the the sequences of the 106 genes. The resulting tree had 100% bootstrap support for each of its branches, and the tree topology is shown in Figure 4.

Further, to study various properties of the MDC criterion and our exact solutions, we ran the methods on synthetic data. To generate those data, we used the Mesquite [12] “Uniform Speciation” (Yule) module to generate 30 species trees, each with 8 taxa and total depth of 1,000,000 generations. Next, within the branches of each of these 30 species trees, 2000 gene trees were simulated under the colaescent process by using the Mesquite module “Coalescence Contained within Current Tree”. The effective population size *N_e_* is 100,000. These were the parameters also recommended by [10]. Finally, to enable studying the statistical consistency of methods, we simulated sampling of gene trees as follows: for each set of 2000 genes trees simulated within the branches of a species tree, we created random samples of 5, 10, 25, 50, 100, 250, 500, 1000, 1500 and 2000 trees; to obtain statistically significant results, we created 30 data sets for each sample size and averaged the results. It is worth mentioning that the parameters we used here, following [10], produced a considerable amount of gene tree incongruence that was similar to the patterns we observed in the two biological data sets.

We have implemented our methods in the PhyloNet software package [11] and analyzed the biological and synthetic data described above by inferring the species tree from the gene trees. In the case of the biological data, and since the “true” species tree is unknown, we compared the species tree inferred by our method to that hypothesized by the authors. We compared the species tree inferred by our method to the one reported in [4] and shown in Figure 3 in the case of the *Apicomplexan* data set, and to the one reported in [1] and shown in Figure 4 for the yeast data set. It is worth mentioning that the species tree inferred by Rokas *et al.* for the yeast data set was also inferred by the BEST Bayesian method [8] and reported in [7].

Since the species tree is known for the synthetic data, we studied the performance of methods by comparing the species tree they inferred against the true species tree. For this comparison, we used the normalized Robinson-Foulds (RF) measure [13], which quantifies the average proportion of branches present in one, but not both, of the trees. A value of 0 for the RF distance indicates the two trees are identical, and a value of 1 indicates the two trees and completely different (they disagree on every branch).

### Analysis of the Apicomplexan Data Set

**Figure 5:**
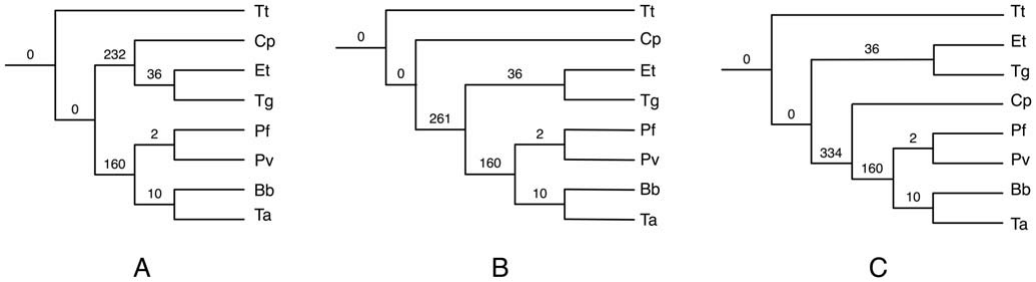
Optimal and sub-optimal trees inferred under the MDC criterion for the *Apicomplexan* data set. **A** The optimal (species) tree inferred by our method for the *Apicomplexan* data set; this tree requires 440 deep coalescences to reconcile all 268 gene trees. The two sub-optimal species trees with 469 and 542 deep coalescences are shown in **B** and **C**, respectively. The value on each branch is the numbers of extra lineages within that branch, when reconciling all 268 gene trees.

Applying our method to the *Apicomplexan* data set, by using the 268 gene trees reported by [4], there was a single optimal tree, which is shown in Figure 5A. The inferred tree requires in total 440 extra lineages to reconcile all 268 gene trees. This tree differs from the tree reported in [4], and shown in Figure 3, with respect to only the single clade (*Cp*, (*Et*, *Tg*)). As Figure 3 shows, the tree reported by Kuo *et al.* places *Cp* as a sibling of the clade ((*Et*, *Tg*), ((*Pf*, *Pv*), (*Bb*, *Ta*))). However, it is important to note that as the authors reported, this placement of *Cp* has very low bootstrap support values of 38, 42, and 40 based on maximum likelihood, maximum parsimony and neighbor joining methods, respectively. Therefore, this grouping is not well-supported, even though both the concatenation and majority consensus methods compute it. Our method differed by placing *Cp* as a sibling of the clade (*Et*, *Tg*). In fact, this grouping was advocated by [14].

**Figure 6:**
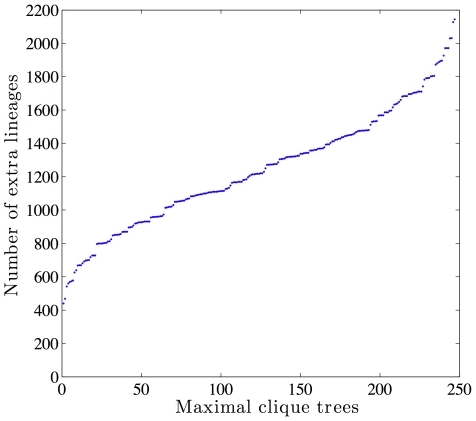
Plot of the number of extra lineages for each of the binary (fully resolved) 247 species tree candidates identified as maximal cliques in the compatibility graph of the gene tree clusters. The first three lowest values are 440, 469 and 542. The trees corresponding to these numbers are shown in Figure 5, respectively.

To investigate this data set further, and particularly the placement of *Cp*, we employed our method in an exploratory mode: the method identified all maximal cliques in the compatibility graph of this data set, and for each maximal clique it computed the optimal fitting of all gene trees by minimizing the deep coalescences. The compatibility graph has 37 vertices (which means there are 37 different clusters induced by all gene trees) and 297 edges. In this graph, there are 247 maximal cliques, all of which have 6 vertices. This allows us to construct 247 fully binary species tree candidates. Figure 6 plots the number of extra lineages for all 247 species tree candidates, sorted from the lowest (which is the optimal one with 440 extra lineages) to the least optimal, which is a maximal clique requiring about 2200 extra lineages to reconcile all gene trees.

We observed that next to the optimal maximal clique with 440 extra lineages, the next two sub-optimal maximal cliques within 100 lineage counts from the optimal one had 469 and 542 extra lineages, respectively. In other words, in addition to the optimal maximal clique, whose corresponding species tree is shown in Figure 5A, there were two additional trees very close in terms of the optimality of MDC. These two trees are shown in Figure 5B and 5C. It is worth noting that the tree in Figure 5B is exactly the tree reported in [4], and that the tree in Figure 5C is the third way to group *Cp*, (*Et*, *Tb*) and ((*Bb*, *Ta*), (*Pf*, *Pv*)). In other words, while our method identified a single optimal tree, this tree along with the two close sub-optimal trees differ from each other by the placement of *Cp*. This fact is already reflected in the community by having two different hypotheses about this placement reported by Levine [14] and Kuo *et al.* [4]. The MDC criterion, however, supports Levine's hypothesis of the species tree, which has 29 fewer deep coalescence events than that proposed by Kuo *et al.*

Beside the biological significance of the results, this analysis highlights several strengths of our method. It is very fast, and it can be run in an exploratory way, which allows the biologist to investigate multiple hypotheses that, while not all optimal, are very close in terms of the optimality criterion. Our method took a few seconds to compute all the values reported in Figure 6. In other words, the method took a few seconds for 247 inferences of species tree candidates, each inference entailing the analysis of 268 gene trees. Second, while the majority consensus method reports a single tree, our method, when run in an exploratory manner, allows for exploring the “landscape” of the different tree topologies that could be species tree candidates. Third, the computation carried out very efficiently using our formulation allows us to explore the number of extra lineages on each of the branches of the inferred species tree, or any candidate tree that the biologist may wish to explore. For example, these numbers for the top three trees are shown on the branches of the trees in Figure 5. Notice that across all three trees, only the number on one branch changes, and that is affected by the placement of *Cp*. These numbers may be useful in a further analysis aimed at estimating divergence times or population sizes, since these two parameters affect the number of extra lineages.

#### Horizontal gene transfer or error in the gene trees?

In our paper, we consider the problem of species tree inference when species/gene tree incongruence is due to incomplete lineage sorting. However, gene trees can also differ from their containing species tree because of horizontal gene transfer (HGT). The phylogeny-based approach to detecting HGT is to reconcile the topological incongruence between a pair of species and gene trees. Here, we argue that we can also make HGT inference based on the number of extra lineages in each branch of the inferred species tree.

Suppose the number of extra lineages in branch *e* = (*u*, *v*) of the species tree *T*′ is small, relative to the size of cluster *C_T_*_′_(*v*). This implies that for most gene trees, lineages in *C_T_*_′_(*v*) coalesce at branch *e* or at branches below *e*. For a few remaining gene trees, there exist extra lineages in *e* that coalesce deeper with other lineages outside *C_T_*_′_(*v*). Given the fact that the deeper a coalescence event, the less likely it will occur [9], we can be more inclined to claim that for those gene trees, their incongruence with the species tree *T*′ on the cluster *C_T_*_′_(*v*) is due to HGT.

**Figure 7:**
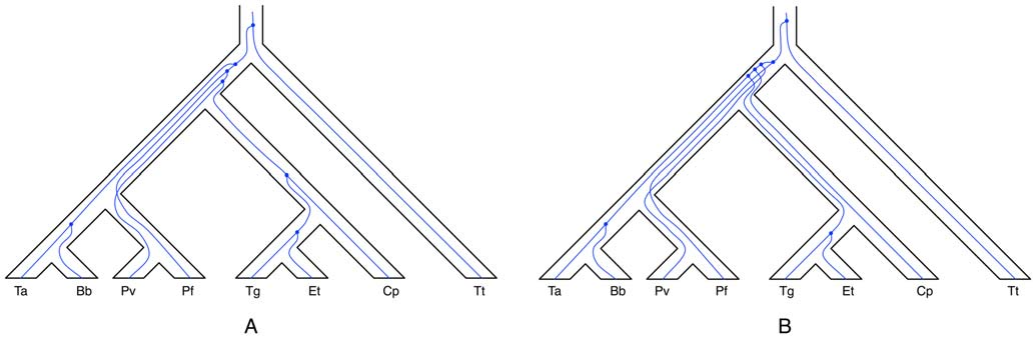
The only two gene trees of the *Apicomplexan* data set that do not have the cluster (*Pv*, *Pf*). **A** The coalescence process, as inferred by MDC, for gene tree (((*Ta*, *Bb*), ((((*Tg*, *Et*), *Cp*), *Pv*), *Pf*)), *Tt*). **B** The coalescence process, as inferred by MDC, for gene tree ((((((*Ta*, *Bb*), (*Tg*, *Et*)), *Cp*), *Pv*), *Pf*), *Tt*).

In Figure 5A, the number of extra lineages in the branch that induces (*Pv*, *Pf*) is 2. This data set has 268 genes, and this means that 266 genes coalesced on the branch incoming into the most recent common ancestor (MRCA) of (*Pv*, *Pf*), while only two gene trees give rise to deep coalescences of the genes in *Pv* and *Pf*. The amount of deep coalescences persisting through a species tree branch may give an indication as to the length (time) and width (population size) of that branch. In this case, having only two out of 268 genes fail to coalesce indicate that the branch incoming into the MRCA of (*Pv*, *Pf*) in the species tree is very long so that the probability of two genes from *Pv* and *Pf* failing to coalesce is small. Therefore, one can zoom in on those two genes and try to understand why they failed to coalesce. The two gene trees are depicted within the branches of the species tree in Figure 7.

**Figure 8:**
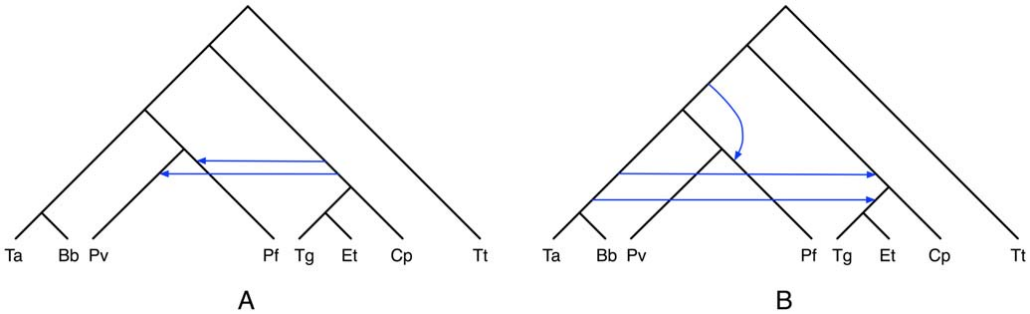
Reconciliations of the two gene trees in Figure 7 and the species tree in Figure 5A assuming HGT as the source of incongruence. **A** The reconciliation scenario for the gene tree (((*Ta*, *Bb*), ((((*Tg*, *Et*), *Cp*), *Pv*), *Pf*)), *Tt*). **B** The reconciliation for the gene tree ((((((*Ta*, *Bb*), (*Tg*, *Et*)), *Cp*), *Pv*), *Pf*), *Tt*).

While deep coalescence may still be a possibility, though one with low probability, two other factors could have given rise to this scenario. One scenario is that in these two gene trees, *Pf* and *Pv* were grouped incorrectly; that is, the gene trees are incorrect. This is an illustration of how simultaneous reconstruction of the species and gene trees may help identify more accurate gene trees. The second scenario involves horizontal gene transfer. In this case, the two gene trees can be reanalyzed, after the species tree has been inferred, so as to reconcile them with the species tree, assuming HGT as a cause of the incongruence. Figure 8 shows the results of such an analysis using the RIATA-HGT method [15] as implemented in PhyloNet [11]. In other words, using this exploratory analysis, which can be rapidly carried out given our method's efficiency, three hypotheses can be generated: one involving deep coalscences, one involving inaccuracy in the gene trees, and a third involving HGT. While tests such as bootstrapping can help assess, to some degree, the support of gene tree branches, some preliminary work has been done on stochastically distinguishing between lineage sorting and horizontal gene transfer (or reticulate evolution in general) as possible causes of incongruence [16],[17].

### Analysis of the Yeast Data Set

The yeast data set contains 106 genes from eight species, with massive discordance among the gene trees, as reported in [1]. The authors concatenated all gene sequences and used maximum likelihood and maximum parsimony methods to reconstruct the species tree, and produced a species tree all of whose branches had 100% bootstrap support; this tree is shown in Figure 4.

**Figure 9:**
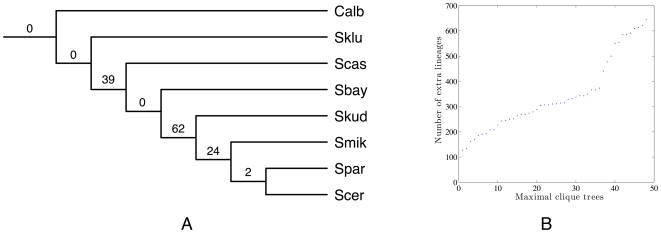
The species tree inferred by our method for the yeast data set. **A** The tree topology and the number of extra lineages, under the optimal reconciliation, for each of its branches. **B** Plot of the number of extra lineages for all 48 species tree candidates.

For our analysis, we reconstructed the gene trees using a maximum parsimony heuristic, and used our method to infer the species tree. There was a single optimal tree found by our method, which is shown in Figure 9A. Clearly, the tree is identical to the one reported by [1]. This tree requires 127 extra lineages to reconcile all 106 gene trees. Edwards *et. al.* [7] also reported the same species tree using their Bayesian tool, BEST [8]. However, while our method took a fraction of a second to infer this species tree, the BEST tool took 800 hours.

**Figure 10:**
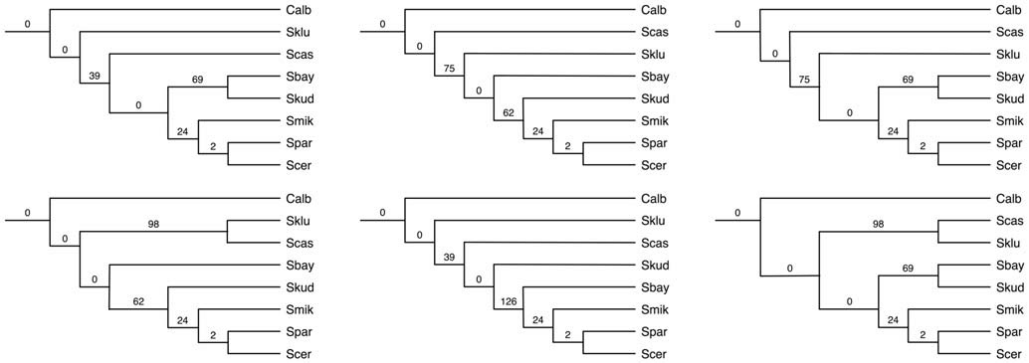
The six best sub-optimal trees for the yeast data set. These trees, from left to right and top down, have in total 134, 163, 170, 186, 191 and 193 extra lineages. The values on the branches are the numbers of extra lineages within them.

As we did with the *Apicomplexan* data set, we also generated all species tree candidates from the compatibility graph built from gene trees (see Methods). The compatibility graph for this yeast data has 17 vertices and 94 edges. We then built 48 binary trees from the 48 maximal cliques in the compatibility graph, and scored the minimum number of deep coalescences required to reconcile all gene trees with each of the trees; these values are shown in Figure 9B. The majority of those species tree candidates require more than 200 extra lineages. The first seven best trees have 127, 134, 163, 170, 186, 191 and 193, respectively. The best tree (the one with 127 extra lineages) is the one shown in Figure 9A, while the other six are shown in Figure 10. A very important point to make here is that these seven trees, while produced by our non-parametric method, include all six maximum posterior probability trees found by BEST in [7]. As before, this exploratory nature of our method allows us to investigate all seven of these trees, not only in terms of their topological differences, but also the implications that selecting one of them has on the reconciliation scenarios of the gene trees in the data set.

### Analysis of the Synthetic Data

The simulated data allowed us to investigate other aspects of the performance of our method, since the true species tree is known and we could compare the inferences made by our method against the true trees.

**Figure 11:**
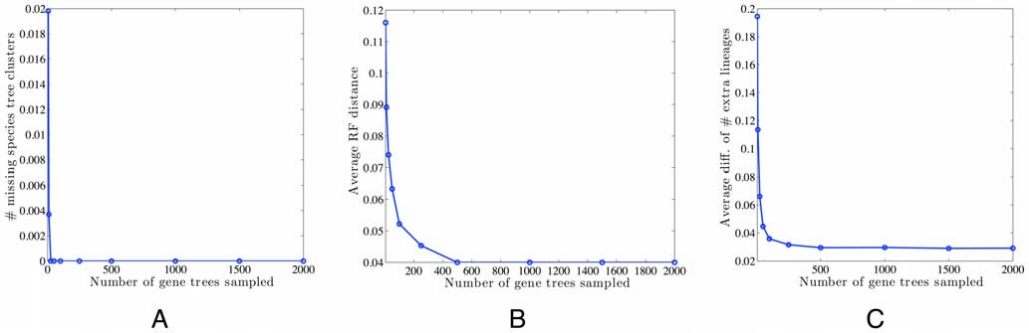
Analysis of the synthetic data sets. **A** The average percentage of clusters induced by species trees that are not found in the set of clusters induced by gene trees. The *x*-axis indicates the number of sampled gene trees. The results are based on the simulated data. **B** The performance of our method on the simulated data. The *x*-axis indicates the number of sampled gene trees. The *y*-axis is the average Robinson-Foulds distance between the species tree and the tree inferred by our method.**C** The difference in the number of extra lineages of the true species tree and that number for the inferred optimal tree.

One of the questions we sought to investigate is whether we need to use the compatibility graph of all species clusters or whether it is sufficient to focus on the compatibility graph of the gene trees. For *n* taxa, there are 2*^n^*−1 clusters (including clusters that have a single taxon and the cluster that contains all taxa, but excluding the “empty cluster”); hence, the compatibility graph of all clusters will have 2*^n^*−1 clusters, which becomes prohibitive for large values of *n*. The number of clusters exhibited by the gene trees, on the other hand, may be much smaller than 2*^n^*−1 in practice. Indeed, this is what we observed in the case of the *Apicomplexian* and yeast data sets. For both data sets, we have *n* = 8 (the number of species), which means the number of all species clusters is 2^8^−1 = 255. However, the numbers of clusters exhibited by the gene trees were 37 and 17, for the *Apicomplexan* and yeast data sets, respectively. This led to drastic reductions in actual running times. Further, this reduction was achieved without compromising the accuracy, as the optimal trees for both data sets were found in the compatibility graphs of the gene trees. To investigate this question further, we analyzed the synthetic data sets with respect to varying the sizes of gene tree samples (see section Data above). For each sample of gene trees, we built the compatibility graph and tested whether the species tree is one of the maximal cliques in the graph. However, rather than the binary question of existence/non-existence, we quantified the proportion of branches in the true species tree that are missing from the closest maximal clique in the graph. If this proportion is zero, then the species tree is one of the maximal cliques. Figure 11A shows the results for this analysis. The results show that when only 25 gene trees are sampled, the compatibility graph provides good “coverage” that the true species tree is already one of the maximal cliques. Even for sample sizes 5 and 10, the proportion of true species tree branches missing from the best maximal clique are 0.02 and 0.004, respectively. These are negligible error rates.

Two important observations are in order. First, these results are well-supported under the experimental conditions we used, which are the parameters used by [10]. Investigations of this question under a richer set of parameters is currently under way. In fact, it is safe to state that there will be points in the parameter space under which the species tree may not be a maximal clique, or even a subgraph, of the compatibility graph. Such a scenario occurs, for example, in the “anomaly zone” [5], which is a setting of specific branch lengths under which the most likely gene tree may be different from the species tree. Second, even though under these parameters the true species tree is one of the maximal cliques in the compatibility graph, this does not imply that optimizing the MDC criterion will correctly identify the species tree. To investigate this issue, we ran our method on the data and compared the optimal tree under MDC with the true species tree. The results are shown in Figure 11B.

In phylogenetics, two of the desirable properties of a phylogenetic method are *statistical consistency* and *fast convergence*. A method is statistically consistent if the error rate in its inference converges to 0 as the amount of data increases. In our case, a method is statistically consistent if the RF distance between the tree it infers and the true tree converges to 0 as more genes are sampled. Fast convergence means that not only does the method converge, but it does so “fast”, where “fast” here means “from small sample size of the data.” The results in Figure 11 show that the MDC criterion has very low error rate. It is important to note that while the average RF distance for our method does not go down to zero, even when 2000 gene trees are used, the RF distance is negligible (about 0.04, which virtually amounts to zero wrong branches in the inferred tree). Yet, the interesting observation here is that combining the results of Figures 11A and 11B, we drew the conclusion that the species tree is one of the maximal cliques in the compatibility graph (particularly for samples of size at least 25), yet it is not the one with the minimum number of extra lineages. Figure 11C shows the difference between the number of extra lineages in the true species tree and that number in the tree inferred by our method. Since our method is guaranteed to find the optimal tree in terms of the number of deep coalescens, this difference (when subtracting the latter number from the former) is non-negative. The results in the figure confirmed our hypothesis: in a few cases, the tree that minimizes the number of deep coalescences is not necessarily the true species tree. Instead, in this case, the species tree is sometimes a sub-optimal one. We observed this same issue even with the two biological data sets, particularly the *Apicomplexan* one. We then investigated how sub-optimal the species tree may be. In all cases when the species tree was not the optimal tree, it was either the first or second sub-optimal one. Once again, this matches results of the analysis of the two biological data sets.

It is important to note that in practice only gene sequences are given and gene trees need to be inferred. Error in the inferred gene trees may affect the performance of the method negatively. Under the MDC criterion, error in the inferred gene trees may masquerade as deep coalescence events, but also may “cancel out” some of the incongruence truly caused by deep coalescence. Therefore, extending the simulation study to include inference of the gene trees, rather than assume they are given, is a task we identify for immediate future research. Nonetheless, the analysis of the two biological data sets above includes the inference of the gene trees themselves, and in these two cases, the MDC criterion provides accurate results.

**Figure 12:**
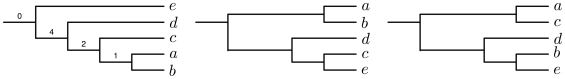
A case in which the optimal tree under the MDC criterion contains at least one cluster that does not occur in any of the input gene trees. Three gene trees over the taxon-set {*a*, *b*, *c*, *d*, *e*}. The tree that minimizes the total number of extra lineages and that consists of only clusters induced by those three trees is the leftmost one. It requires seven extra lineages to reconcile all three gene trees.

**Figure 13:**
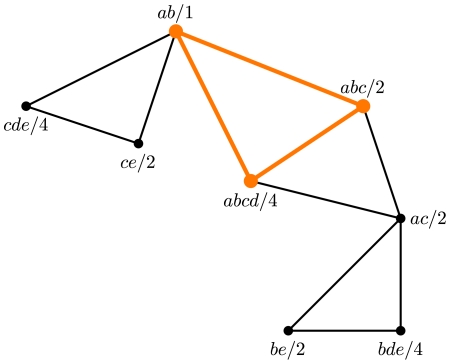
The compatibility graph that is built from clusters induced by the gene trees in Figure 12. Each vertex of the graph corresponds to a cluster (a string next to it), and two vertices are adjacent if the two clusters they represent are compatible. The number following ‘/’ in a vertex label is the total number of extra lineages contributed by the cluster corresponding to that vertex.

**Figure 14:**
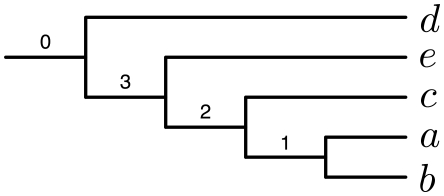
A tree that requires six extra lineages to reconcile the three gene trees in Figure 12.

We finish by showing an example of three gene trees for which the tree that minimizes their deep coalescences is not one of the maximal cliques in the compatibility graph of these three gene trees. Consider the three trees in Figure 12. The compatibility graph that is built from their induced clusters is shown in Figure 13. A minimum vertex-weighted clique of the graph is highlighted with thick lines. Its weight is 1+2+4 = 7, and it corresponds to the leftmost tree in Figure 12. This means that this tree requires seven extra lineages to reconcile the three trees in Figure 12. However, the tree in Figure 14 requires only six extra lineages to reconcile all those three trees. We note that it induces cluster {*a*, *b*, *c*, *e*} that does not appear in any of the three gene trees. This illustrates that in theory the optimal tree under the MDC criterion may not be found in the compatibility graph of the clusters induced by the gene trees.

## Methods

In this section, we describe in detail two methods for reconstructing species trees from multiple gene trees using the MDC criterion. We first introduce notations and definitions necessary for their description.

### Notations

Let *X* be a set of taxa. A phylogenetic tree *T* = (*V*, *E*), where *V* and *E* are its nodes and edges, is a tree with a bijection from *X* to its leaf set 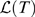. Tree *T* is said to be rooted if the edges in *E* are directed and there is a single internal node *r* with in-degree 0. Except when explicitly stated, in this paper trees are assumed to be rooted and binary. For a node *v*∈*V*, we denote by *T*(*v*) the *clade*, or subtree of *T*, rooted at *v*. The set of leaf labels of *T*(*v*) is called a *cluster*, denoted by *C_T_*(*v*). Cluster *X* and single-element clusters are called trivial. For a cluster *A*, we denote by MRCA*_T_* (*A*) the *most recent common ancestor* (also known as the *least common ancestor*) of taxa in *A* in tree *T*. For two clusters *A*, *B*, we say that they are *compatible* if either *A*⊆*B*, *B*⊆*A* or *A*∩*B* = Ø. Informally, it means that there exists a rooted tree that induces, or contains, both *A* and *B*. A collection of pairwise compatible clusters uniquely defines a rooted tree [18].

### Counting the Number of Extra Lineages

In [9], Maddison introduced the concept of *extra lineages* and a parsimony approach, which we call the “minimize deep coalescences” approach, for species tree inference based on minimizing the number of extra lineages. We first define a mapping between a species tree and gene tree which allows for a precise definition of the number of extra lineages. We then prove that this number can be computed independently for each cluster in the species tree.

Suppose we are given a gene tree *T* and a species tree *T*′ on the same taxon set *X*. We fit tree *T* into *T*′ by mapping each node *v* of *T* according to three rules below:

1. Each taxon (leaf) in *T* is mapped to the corresponding taxon in *T*′.
2. Let *v*′ = MRCA*_T_*_′_(*C_T_*(*v*)), and let *u*′ be the parent node of *v*′. Then, *v* is mapped to any point *p_v_*, excluding node *u*′, in the branch (*u*′, *v*′) in *T*′.
3. If *w* is a proper descendant of *v*, and *w*, *v* are mapped to *p_w_*, *p_v_* in *T*′, then *p_w_* must also be a proper descendant of *p_v_*.

**Figure 15:**
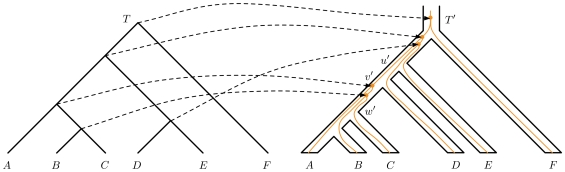
Fitting a gene tree *T* into a species tree *T*′. Here, only mappings of internal nodes of *T* are shown.

Figure 15 shows an example of such a mapping. In this figure, we can see that for branch (*u*′,*v*′) there are two lineages, one being the lineage of the common ancestor of species *A*, *B*, *C*, and one being lineage *D*. In the case where *T* and *T*′ are identical topologically, then we can easily see that there is only one lineage in (*u*′, *v*′), that is one lineage for the common ancestor of *A*, *B*, *C* and *D*. Therefore, for the branch (*u*′, *v*′) in Figure 15, the number of extra lineages is 2−1 = 1. Formally, we define the number of extra lineages in a branch of *T*′ as the number of lineages exiting it minus 1, and the number of extra lineages for *T*′ as the sum of those numbers in all of its branches.

Each *p_v_* in *T*′ that is the image of the mapping of an internal node *v* in *T* is a coalescence event. In Figure 15, there are two coalescence events in branch (*v*′, *w*′), but there are no coalescent events in branch (*u*′, *v*′). We can establish a relationship between the number of extra lineages and the number of coalescence events as follows. Consider a branch (*u*′, *v*′) of *T*′. There are exactly |*C_T_*_′_ (*v*′)| species in the subtree *T*′(*v*′). If there were no coalescence among those species, then there would be |*C_T_*_′_(*v*′)| lineages exiting (*u*′, *v*′). However, each coalescence event merges two lineages into one, and we note that under the mapping's conditions whenever there is a coalescence among lineages from species in *C_T_*_′_(*v*′), it must occur either in a branch of *T*′(*v*′) or in (*u*′, *v*′). Therefore, the actual number of lineages exiting (*u*′, *v*′) is equal to |*C_T_*_′_(*v*′)| minus the total number of coalescence events among species in *T*′(*v*′). We have the following lemma.

**Lemma 1.** *Let n*(*v*′) *be the number of coalescence events occurring among species in C_T_*_′_(*v*′). *Then, the number of extra lineages in branch* (*u*′,*v*′) *is*

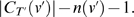
(1)We note that this lemma may not be true without the conditions of the mapping defined above. If we do not have Conditions 2 and 3, then lineages *A*, *B*, and *C* in Figure 15, for example, need not coalesce in branch (*v*′, *w*′). They can coalesce at a branch above *u*′, and in this case there are four lineages (and therefore, three extra ones instead of one) in (*u*′, *v*′).

As we have seen, the number of extra lineages reflects the amount of incongruence between two trees. It is small if two trees are quite similar, and in fact zero if they are identical topologically. Given a set of gene trees, one approach to inferring the species tree is to minimize the number of extra lineages:

**Problem 1** (Species Tree Inference Using the MDC Criterion).

**Input:** *A set of gene trees* 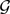.

**Output:** *A tree T such that the total number of extra lineages required to reconcile all gene trees of* 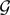 *within T is minimized.*

Let *T* be a gene tree and *T*′ be a species tree. It seems that the number of extra lineages in a branch (*u*′, *v*′) of *T*′ depends on both *T* and *T*′. The following theorem shows it depends only on the gene tree *T* and on the cluster *C_T_*_′_(*v*′). Because of its independence on *T*′, we can denote it by *α*(*C_T_*_′_(*v*′),*T*).

**Theorem 2.** *Let T be a gene tree and T*′ *be a species tree*. *Let* (*u*′, *v*′) *be a branch of the species tree T*′. *Denote by t*_1_, …, *t_k_ all the maximal clades of T such that* 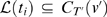 *for* 1≤*i*≤*k*. *Then, the number of extra lineages in* (*u*′, *v*′) *is*

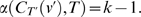
(2)


*Proof*. Consider a clade *t_i_*, 1≤*i*≤*k*. First of all, because *t_i_* is clade of *T* all species in *t_i_* must coalesce into a single lineage (and they must coalesce either in a branch of *T*′ (*v*′) or (*u*′, *v*′) under the mapping's conditions defined above). Second, because *t_i_* is a maximal clade such that 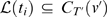, that lineage will not coalesce with any other lineages in *T*′(*v*′) or in branch (*u*′, *v*′) (for otherwise, we will obtain a bigger clade in *T* whose leaf set is still a subset of *C_T_*_′_(*v*′), a contradiction). By Lemma 1, the number of coalescence events occurring among species of *t_i_* is 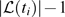. We also note that 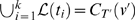. So, by applying this lemma again, we obtain
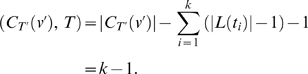
As an example, consider trees *T* and *T*′ in Figure 15. From the figure, we see that there are no extra lineages in branch (*v*′, *w*′). The cluster under *w*′ is {*A*, *B*, *C*}. The clade (*A*, (*B*, *C*)) is a maximal clade of *T* with only species from {*A*, *B*, *C*}. Therefore, the number of extra lineages is 1−1 = 0. On the other hand, consider branch (*u*′, *v*′). There are two maximal clades in *T* with species from {*A*, *B*, *C*, *D*}: (*A*, (*B*, *C*)) and *D*. So, the number of extra lineages in (*u*′, *v*′) is 2−1 = 1.

### Integer-Linear Programming Method

*Linear programming* (LP) is an algorithmic technique for optimizing a *linear* objective function, *cx*, where *c* is a vector of coefficients and *x* is a vector of variables, subject to a set of *linear* constraints *Ax*≤*b*, where *A* is a matrix of coefficients and *b* is a vector of coefficients. This is usually written in the form
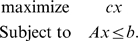
When the variables *x* are required to be integers, the problem becomes an *integer linear programming* (ILP). Solving an ILP problem is NP-hard in general. Nonetheless, software tools for efficiently solving large and hard instances in practice have been developed. One such (commercial) tool is CPLEX, which was developed by the company ILOG (http://www.ilog.com/). In this section, we show how to use ILP to optimize the MDC criterion.

Using Theorem 2, it is possible to compute the number of extra lineages contributed by each individual cluster *without* the need of prior knowledge of the species tree. We can therefore solve Problem 1 exactly by seeking a maximal set of compatible clusters whose total number of extra lineages is minimum. Based on our empirical observation, we find that the species tree is almost always an agglomeration of compatible clusters, each of which appears in at least one of the input gene trees (see Results and Discussion). Based on these two observations, we propose the following method to approximately solve Problem 1:

1. Given a collection 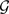 of gene trees, compute the set of nontrivial clusters 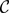 induced by trees in 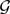;
2. Construct a vertex-weighted compatibility graph *G* based on the set 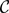; and
3. Find a maximal clique of *G* that minimizes the number of extra lineages, and reconstruct the species tree based on clusters in this clique.

**Figure 16:**
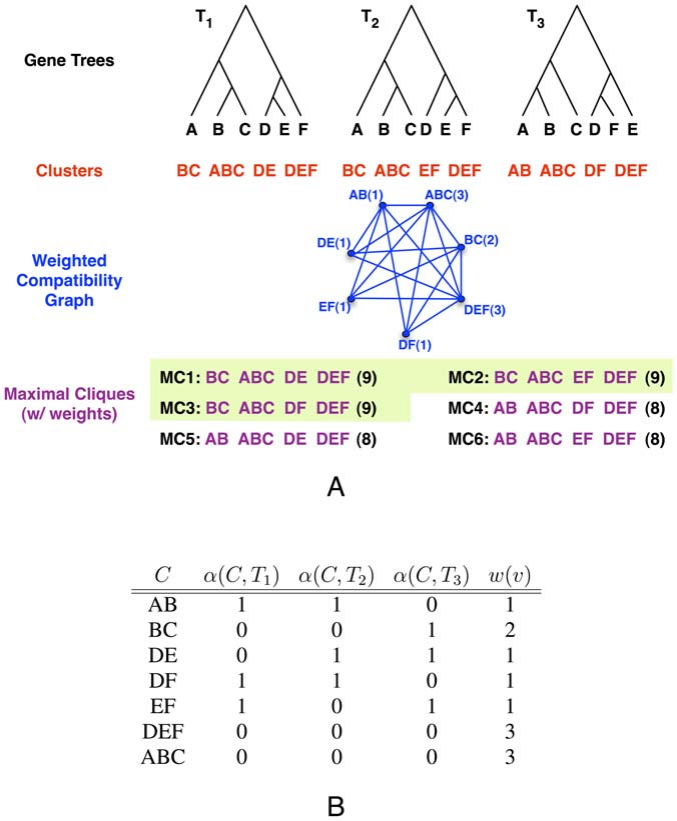
Illustration of our method. **A** A weighted compatibility graph is constructed from the clusters of the input gene trees (*T*_1_, *T*_2_, and *T*_3_). Shown at the bottom are all maximal cliques, along with their weights (the sum of weights of their vertices), of which the three heaviest maximal cliques are highlighted. **B** A table showing the calculation of the weight of each vertex in the compatibility graph, where in each row *v* is the vertex that corresponds to the cluster in that row, and *w*(*v*) = *m*+1−(*α*(*C*, *T*_1_)+*α*(*C*,*T*_2_)+*α*(*C*,*T*_3_)) (*m* = 2 in this case).

We now give the details of the method, using the illustration in Figure 16 as the running example.

#### Constructing the weighted compatibility graph

Given that a collection of pairwise compatible clusters uniquely defines a tree [18], we construct the *compatibility graph G* of all clusters and focus on the cliques in this graph. Let 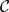 be the collection of all nontrivial clusters of a set 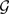. of gene trees. The vertex set of *G* represents clusters in 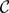. Two vertices are adjacent if the two corresponding clusters are compatible. Since we seek the clique that is simultaneously maximal in terms of size and minimizes the amount of deep coalescence events, we assign weights to the vertices of *G* in a special way. Let *v* be a vertex in the graph *G* and let *A* be the cluster it represents. For each gene tree 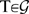., we count the number of extra lineages contributed by *A* as in Eq. (2). In total, cluster *A* contributes 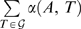 extra lineages. Let *m* be the maximum value of 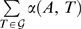 over all 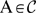. In the example of Figure 16, we have *m* = 2. We assign vertex *v* the weight
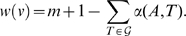
(3)The reason we define *w*(*v*) in this manner, instead of 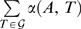, will be clear next, where we describe an efficient ILP formulation for identifying the clique in the compatibility graph that corresponds to a tree that minimizes the total number of deep coalescence events.

#### Finding the tree in the compatibility graph

A clique in the compatibility graph *G* defines a tree, and we seek a clique in *G* such that, on one hand, it has as many vertices as possible (to obtain maximal resolution of the species tree), and on the other hand, the number of extra lineages contributed by its vertices, as defined above, is as small as possible. The way we assign weights to vertices of the compatibility graph *G* allows us to achieve both goals simultaneously.

In the compatibility graph *G*, we will find a maximum vertex-weighted clique. This clique is clearly a maximal one, because each vertex is assigned a positive weight by function *w*(*v*) in Eq. (3), which will guarantee having the maximal number possible of compatible clusters in the species tree. Moreover, because we maximize the clique weight, by the definition of function *w*(*v*), we in fact minimize the total number of extra lineages (among all cliques of the same size).

Finding a maximal vertex-weighted clique in a graph can be converted to a linear programming formulation [19]:
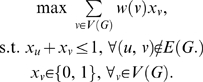
(4)This formulation allows us to solve our problem by using CPLEX. From empirical observations, we find that the compatibility graph *G* is often very sparse. Therefore, the above formulation results in a very large number of constraints *x_u_*+*x_v_*≤1. The following method can reduce the number of constraints to exactly |*V*(*G*)|. For a vertex *u*∈*V*(*G*), let *N*(*u*) be the set of vertices that are adjacent to *u*. The constraint
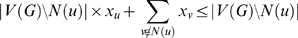
means that if *u* is included in the clique (i.e, *x_u_* = 1), then no vertices in *G* that are not adjacent to *u* are included in the clique (all *x_v_*'s not in *N*(*u*) are 0), and that if any of those vertices is included in the clique, then *u* cannot be in the clique (i.e., *x_u_* must be 0). Therefore, the above linear programming formulation is equivalent to
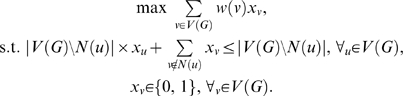
(5)


### Dynamic Programming Method

We can find the optimal species tree without the need to find a maximum vertex-weighted clique in the compatibility graph *G* by employing dynamic programming. Dynamic programming (DP) is a divide-and-conquer algorithmic technique that breaks a problem into sub-problems, solves the sub-problems, and then uses those solutions in an efficient way to form the solution to the main problem. For a problem to be amenable to a DP solution, it has to exhibit certain properties. For more details, the reader is referred to any standard textbook on algorithms; e.g., [20]. We now describe how to solve the MDC optimization problem using a DP algorithm.

Let *t*′ be a rooted binary phylogenetic tree on a fixed taxon subset 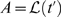 of *X*. Given a collection 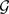 of gene trees, let us denote 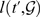 the sum of 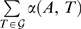 for all clusters *B* in *t*′, including *A*. Further, let 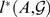 be the minimum value of 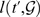 over all possible binary trees *t*′ on *A*. If 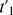 and 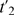 are the two subtrees whose roots are the children of *t*′, then clearly we have
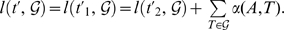
The quantity 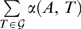 is fixed for each *A*, and therefore, if *t*′ is an optimal tree on *A* such that 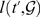 is minimum, then 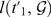 and 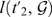 must also be minimum. This allows us to compute 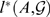 recursively as follows.

1. Let 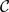 be a collection of nontrivial clusters induced by trees in 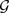 plus cluster *X* and all single-element clusters. We partition 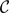 into subsets 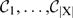, where 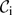, 1≤*i*≤|*X*|, is the collection of all clusters of size *i* in 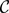.
2. For every 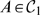, 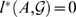, and for 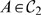, 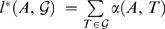.
3. For 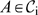, 3≤*i*≤|*X*|,
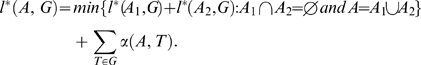

4. Return 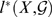.

Although the algorithm described above only returns the number of extra lineages, we can easily modify it so that we can actually reconstruct the optimal species tree. For each *i*, 3≤*i*≤|*X*|, in Step 3, we also record two pointers to optimal subclusters *A*_1_ and *A*_2_. By backtracking those pointers starting with cluster *X*, we can obtain the optimal set of compatible clusters.

Any tree 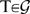 induces exactly |*X*|−2 nontrivial clusters. Therefore, 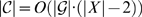. For every *A*⊆*X*, there are at most 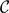 subsets of *A* to look at, and hence Step 3 is executed at most 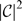 times. The running time of the algorithm is then 
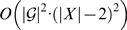
.

The collection 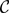 described in the algorithm only contains clusters induced by gene trees in 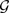. However, we can replace it by the collection of all nonempty subsets of *X* (there are 2^|*X*|^−1 such subsets). In this case, the running time of the algorithm is bounded by 
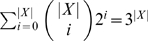
. Although it is exponential, it is significantly better than a brute-force approach that examines all (2|*X*|−3)!! binary rooted phylogenetic trees on *X*.
